# Examining gender, BMI, and lifestyle factors as indicators of type 2 diabetes risk in the Ukrainian population

**DOI:** 10.1007/s12020-025-04353-1

**Published:** 2025-07-16

**Authors:** Jeongwon Richter, Johanna Buchcik, Adekunle Adedeji

**Affiliations:** 1 Department of Health Sciences, Hamburg University of Applied Sciences, Hamburg, Germany; 2 Department of Social Work, Hamburg University of Applied Sciences, Hamburg, Germany; 3 Department of Medical Psychology, University Medical Center, Hamburg-Eppendorf, Hamburg, Germany

**Keywords:** Ukraine, Type 2 Diabetes, Gender, BMI, Self-Reported Risk Factors, Overweight

## Abstract

**Purpose:**

Despite significant advances in diabetes understanding and management, it remains a major global public health challenge. This study examines the influence of gender, body mass index (BMI), and lifestyle factors on developing type 2 diabetes (T2D) in the Ukrainian population. By exploring these key risk factors, the study aims to enhance understanding of T2D determinants and contribute to targeted prevention strategies in Ukraine.

**Methods:**

This current analysis uses cross-sectional secondary data from 12,092 individuals who visited medical mobile teams (MMTs) in four Ukrainian regions (Lviv, Rivne, Dnipro, and Poltava) between April 6 and August 8, 2023. Multiple logistic regression was employed to explore various risk factors associated with T2D.

**Results:**

The results suggest that overweight individuals were 3.02 times more likely to have T2D (Wald (1) = 233.10, p < 0.001, 95% CI: 2.62–3.48), while each unit increase in BMI raised the likelihood by 1.05-fold (Wald (1) = 56.26, p < 0.001). An unhealthy diet increased the risk by 1.29 times (Wald (1) = 13.99, p < 0.001). Women had 1.16 times higher T2D prevalence than men (Wald (1) = 3.93, p <0.05). Sedentary individuals were 16.1% less likely to have T2D (Wald (1) = 8.69, p < 0.01).

**Conclusion:**

This study is significant in that self-perceived overweight status was found to be the most important risk factor associated with T2D prevalence. This provides important guidance for organisations seeking to support T2D diagnosis and management efforts in Ukraine and emphasises the need to recognise self-perceived overweight status as an important consideration.

## Introduction

Diabetes represents a significant global health challenge. According to the International Diabetes Federation (IDF) Atlas 2021, an estimated 537 million people will live with diabetes in 2021, rising to 783 million by 2045 [1]. Despite substantial advances in understanding and managing diabetes, it remains a major global public health challenge of the 21st century. There were 6.7 million deaths from diabetes-related causes among people aged 20 to 79 years in 2021. Still, it also resulted in significant healthcare expenditures, accounting for an estimated $966 billion of global healthcare spending in 2021, an increase of 316% over the past 15 years [1].

Several factors have been identified as contributors to the prevalence and management of T2D. These include biological attributes such as *gender*, *body mass index* (BMI), and *lifestyle factors* like diet, physical activity, smoking, and alcohol consumption. Gender differences in T2D are particularly striking, with men more likely to be diagnosed at younger ages and possessing lower body fat percentages, whereas women tend to have higher obesity prevalence [2]. In addition, BMI and overweight status are critical risk factors for T2D, as evidenced by studies conducted in Ukraine, East Asia, and other regions, which consistently show a strong association between elevated BMI and increased disease risk [3–6].

*Lifestyle behaviours* play an essential role in the prevention and management of T2D. Diet quality is a key determinant, with unhealthy eating patterns, such as high consumption of red and processed meats, sugar-sweetened beverages, and excessive salt intake, linked to increased risk. Conversely, diets rich in whole grains, fruits, vegetables, and low-fat dairy products have been shown to reduce T2D risk significantly [7, 8]. Physical activity, especially moderate to vigorous exercise, is another proven factor that reduces T2D incidence and improves glycemic control [9, 10]. Smoking consistently elevates T2D risk due to its negative effects on insulin sensitivity and systemic inflammation [11]. The relationship between alcohol consumption and T2D is more nuanced, with moderate consumption demonstrating a protective effect, while excessive and binge drinking significantly heightens risk [12, 13].

The specific context of *Ukraine* offers a distinct and critical context for studying Type 2 diabetes (T2D) risk factors, given its unique population health profile and the compounded impacts of the ongoing war. The country faces a high prevalence of T2D risk factors, including a national average BMI of 26.8 kg/m^2^ and widespread obesity, particularly among women [14]. Smoking and alcohol consumption are notably prevalent, with significant gender disparities - 50.3% of men smoke compared to 16.7% of women, and heavy drinking is common among men [15]. Poor dietary habits, such as low fruit and vegetable intake and excessive salt consumption, further compound these risks. Interestingly, Ukraine demonstrates relatively high physical activity levels, with only 10.0% failing to meet World Health Organization (WHO) recommendations, underscoring the unique interplay of protective and harmful behaviors in this population [14].

The ongoing war in Ukraine has exacerbated these challenges, introducing additional stressors such as displacement, food insecurity, and limited access to healthcare. These factors likely worsen diet quality, increase sedentary behavior, and heighten stress levels, all of which contribute to T2D development. Mobile medical teams (MMTs), operating as critical healthcare providers during the crisis, offer a unique opportunity to analyse real-time data on health outcomes in conflict-affected settings.

By investigating the roles of gender, BMI, and lifestyle factors in T2D risk among Ukrainians served by MMTs, this research addresses a critical gap in understanding the intersection of chronic disease risk and humanitarian crises. Its findings will inform tailored, context-sensitive interventions to improve diabetes prevention and management in Ukraine and similar regions under crisis. By focusing on these associations, this research endeavors to address the following specific research objectives:

1. To determine the prevalence rate of T2D among the Ukrainian population who visited mobile medical teams.
2. To identify the differences in the distribution of T2D risk factors between diabetics and non-diabetics.
3. To analyse the predictive effects of identified risk factors on the incidence of T2D.

## Methods

### Study design and data source

This study utilised cross-sectional secondary data sourced from four regions of Ukraine: Lviv, Rivne, Dnipro, and Poltava. The data used in the study was collected from the 6th of April till the 8th of August 2023. The Act4Health project by GFA Consulting Group GmbH was funded by the Swiss Development and Cooperation (SDC). The project team at the GFA planned and designed the primary data collection procedures, which ensured compliance with ethical guidelines and obtained informed consent from the participating patients. In response to the humanitarian crisis in Ukraine, nine MMTs have been deployed to deliver healthcare services. These teams are composed of multi-disciplinary health professionals, including medical doctors (MDs), nurses, psychologists, and drivers. Electrocardiogram (ECG) screening, diagnosis, care management, laboratory testing, counselling, first aid, information distribution, and health advice were provided during their visit. Data acquisition for this study was employed by using the ArcGIS survey 123digital tool. In total, 18,589 data points were collected in this study.

**Fig. 1:**
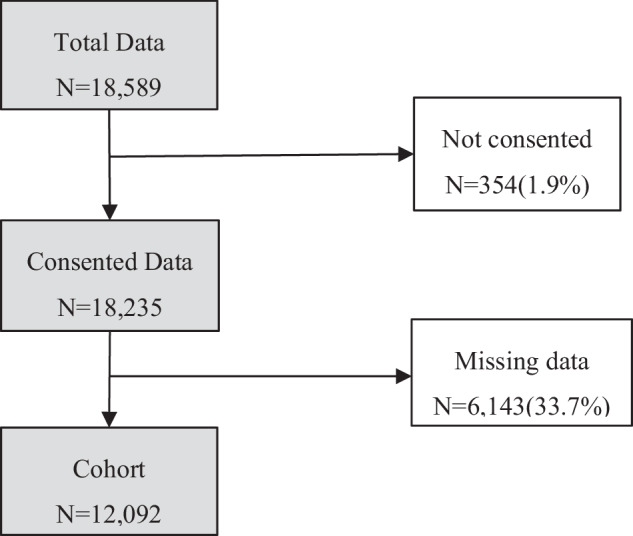
Eligibility criteria for research

From these, (i) non-consent data and (ii) missing data in the variables including age, height, and weight were removed (see Fig. 1). After applying the exclusion criteria, 12,092 subjects were eligible for analysis.

### Measures

#### Lifestyle factors

Lifestyle factors assessed in this study included smoking, alcohol use, sedentary behaviour, being overweight, and unhealthy eating habits. Patients provided self-reports of these factors during an initial consultation with the MMT. The questionnaire used in this consultation was designed to comprehensively assess participants’ lifestyle choices and health behaviours. Clear and straightforward language was used to make it easy for participants to understand and respond. During the data collection process, specific definitions or criteria were not pre-defined by the data providers, and responses were collected in a binary format. Lifestyle factors were coded dichotomously as ‘no’ (0) or ‘yes’ (1), indicating the presence or absence of each risk factor. *Example Question: “Do you currently smoke cigarettes, cigars, or use any form of tobacco?”*

#### Risk score

The risk score is calculated by summing participant’s responses to various risk factors as assessed in the questionnaire. These factors included smoking, alcohol consumption, sedentary behaviour, overweight, and unhealthy diet. Each factor is given a score ranging from 0 (indicating none of the mentioned behaviours) to 5 (indicating the presence of all the mentioned behaviours). Lower scores indicated a self-perceived healthier lifestyle status, while higher scores indicated a self-perceived unhealthier lifestyle status. This scoring system facilitated the quantification of participants’ overall risk level based on their reported behaviours. The risk score was created specifically for the present dataset to provide an intuitive indicator of lifestyle risk in a conflict setting. The score showed acceptable internal discrimination for prevalent T2D in our sample (AUC = 0.65, 95% CI 0.64–0.67).

#### Disease determination

If patients visiting the MMTs were overweight or obese, they were screened by a physician. T2D was defined as ${random\; blood\; glucose}\ge 11.1{mmol}/L(200{mg}/{dl})$ [16] and included patients who were taking diabetes medication or self-reported. The dependent variable, T2D, was encoded as ‘no’ (0) and ‘yes’ (1).

#### BMI and weight group

BMI was calculated using self-reported height and weight. The formula is ${\boldsymbol{BMI}}={\boldsymbol{kg}}/{\boldsymbol{m}}{\boldsymbol{2}}$.

The group is divided into 4 different nutritional status categories based on WHO criteria [17].

$${BMI} < 18.5{kg}/{m}^{2}={\rm{Underweight}}$$

$$18.5{kg}/{m}^{2}\le {BMI} < 25{kg}/{m}^{2}={\rm{Normal\; weight}}$$

$$25{kg}/{m}^{2}\le {BMI} < 30{kg}/{m}^{2}={\rm{Overweight}}$$

$${BMI}\ge 30{kg}/{m}^{2}={\rm{Obese}}$$

#### Sociodemographic data

This study uses sociodemographic data to provide a comprehensive understanding of the participants and the analysis. The variables used include gender (female, male), age, age group and migration status (local, internally displaced person (IDPs)).

### Statistical analysis

For this study, all statistical analyses were conducted using Statistical Package for the Social Science (SPSS) version 29 (IBM Corp., Armonk, NY). Descriptive statistics were performed for each indicator and sociodemographic variable to understand the basic features of the data disaggregated by gender and T2D prevalence. Independent samples t-tests were performed to compare BMI and lifestyle score by gender and prevalence of T2D. Multiple logistic regression was computed to examine which risk factor variables affect T2D. All variables except BMI were treated as binomial. The dataset included 12,092 in the analyses. Logistic regression models were constructed with T2D as the dependent variable and gender, BMI, overweight, unhealthy diet, sedentary lifestyle, alcohol consumption and smoking as independent variables. Significance levels were denoted as follows: p < 0.05 (significant), p < 0.01 (highly significant), p < 0.001 (extremely significant).

## Results

### Baseline demographics

The analysed sample comprised 12,092 participants. With a mean age of 59.75 (SD = 15.88) years, 72.7% were female and the largest proportion were in their sixties and seventies. Women were more likely to be classified as overweight or obese. For both men and women, the obese, normal weight category for women and the normal weight, obese category for men were the most common weight groups after overweight. While objective measure classifies 70.2% of women and 63.9% of men as overweight, only 47.0% of women and 36.8% of men described themselves as overweight. Men were significantly more likely to smoke and drink alcohol than women, and more than half of both sexes reported having unhealthy eating habits (63.6% in females and 67.6% in males respectively) (see Table 1).

**Table 1:** Characteristics of the participants in Ukraine (n = 12,092)

Variables		Both Sexes		Female		Male	
		n	%	n	%	N	%
Total(n)		12,092	100.0	8793	72.7	3299	27.3
T2D	No	10,506	86.9	7556	85.9	2950	89.4
	Yes	1586	13.1	1237	14.1	349	10.6
Migration Status	Local	11,327	93.7	8186	93.1	3141	95.2
	IDPs	765	6.3	607	6.9	158	4.8
Age		59.75 ± 15.88		60.55 ± 15.22		57.59 ± 17.33	
Age group	Under 10	74	0.6	33	0.4	41	1.2
	11 ~ 20	292	2.4	151	1.7	141	4.3
	21 ~ 30	363	3.0	244	2.8	119	3.6
	31 ~ 40	775	6.4	554	6.3	221	6.7
	41 ~ 50	1361	11.3	951	10.8	410	12.4
	51 ~ 60	2316	19.2	1712	19.5	604	18.3
	61 ~ 70	3730	30.8	2757	31.4	973	29.5
	71 ~ 80	2554	21.1	1899	21.6	655	19.9
	81 over	627	5.2	492	5.6	135	4.1
BMI		27.89 ± 5.28		28.22 ± 5.46		27.04 ± 4.60	
Weight group	Underweight	207	1.7	145	1.6	62	1.9
	Normal	3606	29.8	2479	28.2	1127	34.2
	Overweight	4477	37.0	3130	35.6	1347	40.8
	Obese	3802	31.4	3039	34.6	763	23.1
Smoking	No	10,692	88.4	8506	96.7	2186	66.3
	Yes	1400	11.6	287	3.3	1113	33.7
Alcohol consumption	No	11,720	96.9	8742	99.4	2978	90.3
	Yes	372	3.1	51	0.6	321	9.7
Unhealthy Diet	No	4272	35.3	3204	36.4	1068	32.4
	Yes	7820	64.7	5589	63.6	2231	67.6
Overweight	No	6743	55.8	4657	53.0	2086	63.2
	Yes	5349	44.2	4136	47.0	1213	36.8
Sedentary Lifestyle	No	6139	50.8	4458	50.7	1681	51.0
	Yes	5953	49.2	4335	49.3	1618	49.0
Risk score		1.73 ± 1.10		1.64 ± 1.06		1.97 ± 1.18	
	0	1999	16.5	1588	23.7	411	12.5
	1	3020	25.0	2276	24.9	744	22.6
	2	3715	30.7	2722	29.2	993	30.1
	3	3032	25.1	2150	21.6	882	26.7
	4	282	2.3	57	0.5	225	6.8
	5	44	0.4	0	0.0	44	1.3

### Characteristics of subjects from diabetic and nondiabetic

The proportion of patients with T2D among all subjects was around 13.1%. People in their 60 s had the highest rate of diabetes, but this is hardly an outlier, as this is also the age group with the highest rate of demographic characteristics. Among diabetics, the prevalence of hypertension and cardiovascular disease was high, at 82.0 and 68.6%, respectively. The proportion of people who were obese and perceived to be overweight was higher in the diabetes group. In addition, the percentage of people who reported having unhealthy eating habits and leading a sedentary lifestyle was higher in the diabetes group than in the nondiabetic group (see Table 2).

**Table 2:** Characteristics of subjects from diabetic and nondiabetic (n = 12,092)

Variables		Diabetic		Nondiabetic	
		n	%	n	%
Total(n)		1586	13.1	10,506	86.9
Gender	Female	1237	78.0	7556	71.9
	Male	349	22.0	2950	28.1
Migration Status	Local	1493	94.1	9834	93.6
	IDPs	93	5.9	672	6.4
Age		65.14 ± 10.02		58.93 ± 16.4	
Age group	Under 10	0	0.0	74	0.7
	11 ~ 20	5	0.3	287	2.7
	21 ~ 30	3	0.2	360	3.4
	31 ~ 40	21	1.3	754	7.2
	41 ~ 50	94	5.9	1267	12.1
	51 ~ 60	331	20.9	1985	18.9
	61 ~ 70	622	39.2	3108	29.6
	71 ~ 80	452	28.5	2102	20.0
	81 over	58	3.7	569	5.4
Hypertension	No	286	18.0	3585	34.1
	Yes	1300	82.0	6921	65.9
CVD	No	498	31.4	3887	37.0
	Yes	1088	68.6	6619	63.0
BMI		30.54 ± 5.0		27.49 ± 5.19	
Weight group	Underweight	1	0.1	206	2.0
	Normal	184	11.6	3422	32.6
	Overweight	576	36.3	3901	37.1
	Obese	825	52.0	2977	28.3
Smoking	No	1442	90.9	9250	88.0
	Yes	144	9.1	1256	12.0
Alcohol consumption	No	1550	97.7	10,170	96.8
	Yes	36	2.3	336	3.2
Unhealthy Diet	No	417	26.3	3855	36.7
	Yes	1169	73.7	6651	63.6
Overweight	No	422	26.6	6321	60.2
	Yes	1164	73.4	4185	39.8
Sedentary Lifestyle	No	764	48.2	5375	51.2
	Yes	822	51.8	5131	48.8
Risk score		2.10 ± 0.94		1.67 ± 1.11	
	0	61	3.8	1938	18.4
	1	370	23.3	2650	25.2
	2	560	35.3	3155	30.0
	3	542	34.2	2490	23.7
	4	46	2.9	236	2.2
	5	7	0.4	37	0.4

### Independent samples t-test

Independent samples t-tests were performed to explore differences in BMI and risk score by gender and prevalence of T2D. For BMI, there were significant differences between gender and T2D prevalence. The results were t = −11.89, p < 0.001 and t = −21.90, p < 0.001 in gender and T2D prevalence respectively. Females had a mean BMI of 28.22, while males had a slightly lower mean BMI of 27.04 highlighting a gender-related variance with females having a higher mean BMI. Furthermore, concerning T2D prevalence, participants without T2D had a mean BMI of 27.49, while those with T2D had a significantly higher mean BMI of 30.54. This difference underlines a significant association between T2D prevalence and elevated BMI levels Table 3.

**Table 3:** BMI differences by gender and T2D prevalence

Variables		BMI			t(p)
		N	Mean	SD	
Gender	Female	8793	28.22	5.46	−11.89(<0.001)***
	Male	3299	27.04	4.60	
T2D	No	10,506	27.49	5.19	−21.90(<0.001)***
	Yes	1586	30.54	5.04	

***p < 0.001

Similarly, significant differences in risk scores were observed based on both gender and T2D prevalence. The calculated values were t = 14.88, p < 0.001 and t = −16.67, p < 0.001 in gender and T2D prevalence respectively. Females had a mean risk score of 1.64, while males had a higher mean score of 1.97, indicating a gender difference with males having a higher mean risk score. In addition, participants without T2D had a mean risk score of 1.67, while those with T2D had a higher mean score of 2.10. This observation suggests a notable association between T2D prevalence and elevated risk scores, implying that participants with T2D tend to have higher risk scores Table 4.

**Table 4:** Risk score differences by gender and T2D prevalence

Variables		Risk Score			t(p)
		N	Mean	SD	
Gender	Female	8793	1.64	1.06	14.88(<0.001)***
	Male	3299	1.97	1.18	
T2D	No	10,506	1.67	1.11	−16.67(<0.001)***
	Yes	1586	2.10	0.94	

***p < 0.001

### Multiple logistic regression analysis

In this study, a multiple logistic regression model was applied to identify predictors that can explain variance in the increasing risk of developing T2D. The Omnibus tests of model coefficients indicated a significant model fit (Chi-square = 719.24, *df* = 7, p < 0.001). The Cox & Snell R^2^ and Nagelkerke R^2^ values were 0.06 and 0.11, respectively, suggesting that the model explains a moderate proportion of the variance in T2D. Our analysis revealed that gender, unhealthy diet, overweight, sedentary lifestyle and BMI were significant predictors of T2D (see Table 5). Of these, being overweight emerged as the most influential factor (Wald (1) = 233.10, p < 0.001). Those who reported being overweight were 3.016 times (95% CI: 2.62–3.48) more likely to have T2D than their non-overweight counterparts. Additionally, a 1 increase in BMI was associated with a 1.046-fold rise in the likelihood of T2D (Wald (1) = 56.26, p < 0.001). Regarding diet, individuals who reported unhealthy eating habits were 1.286 times more likely to have T2D than those who did not (Wald (1) = 13.99, p < 0.001). In terms of gender differences, women were found to have a 1.157 times higher prevalence of T2D than men (Wald (1) = 3.93, p < 0.05). However, the model indicated that sedentary individuals were 16.1% less likely to have T2D compared to active individuals (Wald (1) = 8.69, p < 0.01).

**Table 5:** Coefficients of multiple logistic regression model

Variable	B	S.E.	Wald	df	Sig.	Exp(B)	95% C.I for Exp(B)	
							Lower	Upper
Gender (1)	0.146	0.074	3.929	1	0.047*	1.157	1.002	1.337
Smoking (1)	−0.020	0.107	0.035	1	0.851	0.980	0.794	1.209
Alcohol (1)	−0.123	0.191	0.413	1	0.520	0.884	0.608	1.286
Unhealthy Diet (1)	0.252	0.067	13.998	1	<0.001***	1.286	1.127	1.468
Overweight (1)	1.104	0.072	233.092	1	<0.001***	3.016	2.618	3.476
Sedentary Lifestyle (1)	−0.176	0.060	8.689	1	0.003**	0.839	0.746	0.943
BMI	0.045	0.006	56.255	1	<0.001***	1.046	1.034	1.059
Constant	−5.499	1.004	29.977	1	<0.001	0.004		

Reference group: Gender*Male, Smoking, Alcohol, Unhealthy Diet, Overweight, Sedentary Lifestyle*No

*p < 0.05, **p < 0.01, ***p < 0.001

## Discussion

The objective of this study was to delineate the relationships between socio-economic factors (e.g., gender), health-related factors (e.g., BMI), lifestyle factors (e.g., smoking, alcohol consumption, sedentary lifestyle, overweight, and unhealthy diet), and the prevalence of Type 2 Diabetes (T2D) amidst the humanitarian crises in Ukraine. Results revealed significant associations between T2D and several factors: gender, unhealthy diet, overweight status, sedentary lifestyle, and BMI.

The association between BMI and T2D was consistent with prior studies in Ukraine and globally [3, 5, 6]. However, this study also emphasised the limitations of BMI as a universal indicator, particularly when compared to waist circumference (WC) and waist-to-height ratio (WtHR), which have demonstrated stronger correlations with diabetes risk, especially among women [18, 19]. Interestingly, the study highlighted a gap between objective BMI measurements and self-perceived overweight status, reflecting cultural and age-related differences in weight perception. This observation aligns with findings from another study noting that as individuals get older, even those classified as overweight or obese are more likely to think they are a healthy weight, suggesting that perceptions of ideal weight change with age [20]. Gender differences were also evident with women exhibiting higher rates of T2D and healthier diets, but lower perceived risk scores compared to men. Similar patterns have been observed in studies conducted in South Korea, where women, despite adopting healthier lifestyles, tended to have higher diabetes risk compared to men [6]. Correspondingly in this study, the average risk score was higher for men (1.97) than for women (1.64), consistent with trends observed in the WHO STEPS survey among the Ukrainian population [14].

The current analysis found an unexpected association between physical activity and increased risk of T2D, contrasting with findings from the Whitehall II study, which showed that moderate-to-vigorous exercise reduces T2D risk [10]. This discrepancy may be partly attributed to the unique situation in Ukraine, where physical activity levels are among the highest in the WHO European Region [14]. However, the ongoing war in Ukraine has significantly disrupted daily life, including exercise routines, access to safe outdoor spaces, and mental well-being. Stress and psychological strain associated with the conflict may also undermine the benefits of physical activity, while other factors such as poor diet and socio-economic stress exacerbate T2D risk [21, 22]. Previous research found no significant differences in physical activity levels across weight categories, indicating that even high physical activity may not sufficiently mitigate T2D risk in this context [3]. Further studies are needed to explore how the war’s impact on daily life, lifestyle factors, and physical activity intensity influences T2D risk in this population.

The association between unhealthy diets and T2D prevalence was evident in this study, with those having unhealthy diets at a higher risk of developing T2D. This result is consistent with previous systematic reviews and meta-analyses [7, 8]. Previous reports also indicated that two-thirds of Ukrainians do not consume the recommended amount of fruit and vegetables. In that study, women (40.6%) ate more than five portions of fruit and vegetables per day compared to 26.8% of men [14]. In this study, women (36.4%) reported healthier diets than men (32.4%). However, our study’s self-reported concept of a ‘healthy diet’ remains subjective and varies greatly by age, culture, and geography, as the perception of healthy eating is highly individualized [23]. Future research should aim to establish a more uniform definition of healthy eating to reduce the variability in dietary assessments and better understand its relationship with T2D.

Smoking and alcohol consumption, while commonly associated with T2D in other regions, were not significant factors in this study, possibly due to underreporting or simplified data categorisation. Previous studies in East Asia identified smoking and alcohol consumption as risk factors for T2D prevalence [11, 24]. However, the current findings align with studies using binary (smoking /non-smoking) responses rather than detailed criteria for smoking, which failed to show statistically significant differences [25]. This discrepancy may be attributed to the possibility of underreporting in studies based on self-reporting without specific criteria for smoking. Given the complex relationship between alcohol consumption and T2D risk [12], the current results may explain why it did not show a statistically significant association. This finding may be due to the use of dichotomous responses (drinker/non-drinker) rather than detailed alcohol consumption data per day. Despite the non-significance, the findings indicate that men were more likely than women to smoke and drink alcohol.

Before the current conflict, national surveys had already revealed an unfavourable NCD profile in Ukraine. According to the 2019 WHO STEPS survey, 59.1% of adults were overweight or obese(24.8% obese), 33.9% were daily smokers, 55.6% had consumed alcohol in preceding 30 days, 66.4% consumed fewer than five portions of fruit and vegetables per day, and only 10.0% of adults failed to meet WHO physical- activity recommendations [14]. That survey comprised 4409 adults (women 62.6%, men 37.4%), with 59.0% aged 45–69 years. In our 2023 conflict-period cohort, the prevalence of overweight or obesity increased to 68.4%. Smoking remained high among men (34.0%). Reports of a sedentary lifestyle increased markedly to 49.0%, nearly five times the pre-war figure. Self-reported alcohol use fell to 3.0% overall (men 9.7%, women 0.6%). This apparent decline likely reflects under-reporting or reduced availability rather than a true behaviour change. Our sample was older (mean 59.8 years) and more female (73.0%).

These observations highlight the need for conflict-sensitive prevention and screening strategies while Ukraine continues to face acute humanitarian needs and simultaneously plans for recovery. Priority should be given to systematic diabetes screening of adults who perceive themselves as overweight and to nutrition-focused counselling that helps people reach the fruit-and-vegetable recommendations despite disrupted food systems. Mobile medical teams could also create safe opportunities for regular physical activity in displacement settings.

### Strengths and limitations

The study was conducted in a conflict-affected country during an acute crisis phase and collected extensive data (12,092 cases) on a range of risk behaviours. It represents an important effort to fill a knowledge gap on the epidemiology of diabetes in humanitarian settings. Unlike most existing studies that focus on conflict-affected populations in the Eastern Mediterranean [9], this study targeted areas directly affected by conflict, providing valuable insights into the epidemiology of diabetes in these challenging contexts.

Despite its significant contribution, the findings have several limitations. Firstly, as it relies on self-reported data on risk factors presented in a dichotomous format without detailed criteria, the data might omit relevant information and may be affected by the subjective nature of individual responses. Second, the significantly higher proportion of female participants compared to males may induce bias, which could lead to distorted results and affect the generalisability of the findings to other populations. Third, it is also essential to acknowledge the inherent limitations of cross-sectional studies, which cannot establish causal relationships or examine temporal relationships. Fourth, sampling strategies that focus only on individuals accessing health services through mobile teams in crises may limit the generalisability of findings to the wider population affected by conflict. Finally, the risk score was developed specifically for this dataset and should be regarded as exploratory. It has not yet been externally validated, so its predictive accuracy and calibration are currently unknown. Future longitudinal studies are required to confirm its utility and to elucidate the causal pathways that link lifestyle patterns to T2D in conflict-affected populations.

### Conclusion

This study examines the association between type 2 diabetes (T2D) and several risk factors, including gender, body mass index (BMI), and self-perceived risk factors, including smoking habits, alcohol consumption, unhealthy eating habits, physical inactivity, and overweight, in the context of the humanitarian crisis in Ukraine. The study is significant as self-perceived overweight status was the most important risk factor associated with T2D prevalence. These findings provide important guidance for organisations seeking to support T2D diagnosis and management efforts in Ukraine and highlight the need to recognise self-perceived overweight status as an important consideration.

The findings also suggest a strategic approach for policymakers during and post-crisis to campaign for standardised recommendations for people who perceive themselves to be overweight to visit a clinic for diabetes screening. Such proactive measures aim to improve the early detection and management of T2D, thereby improving patient outcomes.

Further research is needed to establish the bidirectional relationship between CVD, hypertension, and T2D. In Ukraine, CVD stands out as the leading cause of death from NCDs [14]. In addition, CVD is a well-known complication of T2D [26], also, findings from our study show a high prevalence of CVD and hypertension in individuals with T2D. In addition, future studies conducted in the aftermath of the crisis will be invaluable in enriching our understanding of the impact of this crisis on health outcomes through comparative analysis with current findings. This ongoing research will not only broaden our understanding of the multifaceted nature of T2D risk factors but will also inform more effective health policies and interventions in similar situations in Ukraine and around the world.

## Data Availability

The data supporting the findings of this study is available upon reasonable request to the corresponding author.

## List of abbreviations

BMI: Body Mass Index
CVD: Cardiovascular Disease
ECG: Electrocardiogram
IDF: International Diabetes Federation
IDP: Internally Displaced Person
MMT: Mobile Medical Team
NCD: Non-Communicable Disease
PA: Physical Activity
T2D: Type 2 Diabetes
WC: Waist Circumference
WtHR: Waist-to-Height Ratio
